# Time-series dataset of honey bee colony dynamics before, during, and after sunflower pollination

**DOI:** 10.1186/s13104-026-07812-8

**Published:** 2026-04-06

**Authors:** Igor Kurdin, Aleksandra Kurdina

**Affiliations:** 1 Institute of Information Technology, Warsaw University of Life Sciences, Warsaw, Poland; 2 AmoHive, Warsaw, Poland

**Keywords:** Precision beekeeping, Precision agriculture, Crop pollination, Sunflower pollination, Smart hives, IoT, Time series, Data provenance

## Abstract

**Objectives:**

Precision beekeeping is an integral part of precision agriculture, which relies on sensor technologies and high-quality datasets to quantify and optimize ecosystem services such as crop pollination. To support reproducible research and the planning and evaluation of crop pollination campaigns in precision beekeeping, we release a time-series dataset that characterizes colony dynamics before, during, and after pollination, using sunflower as a case study.

**Data description:**

We release synchronized, non-invasive time series from nine smart hives (*Apis mellifera*) monitored in Ukraine (Europe/Kyiv) from 01 May to 31 Aug 2024, including a sunflower pollination service window (07–23 Jul 2024) and a documented attractant intervention. Sensors record hive weight, in-hive and ambient temperature, in-hive and ambient relative humidity, and device signals (processor temperature and stabilized solar voltage). The repository includes raw telemetry exports, cleaned hourly series aligned to a fixed local time grid, and a beekeeper event log, together with reproducible scripts and a documented processing protocol.

## Objective

Commercial pollination by honey bees can increase crop yield and quality, including sunflower [1]. However, real-time monitoring of pollination service delivery remains limited in practice, particularly within short and weather-dependent flowering windows [2]. As a result, pollination outcomes are often evaluated after flowering using agronomic endpoints such as yield, including fruit or seed quantity and quality [3]. Indicators of colony development dynamics in the pre-pollination period are essential because they determine readiness and expected service capacity during pollination [4]. Precision beekeeping technologies can provide non-invasive proxies of colony activity from smart hive telemetry [5, 6]. Despite this progress, publicly available, well-documented, and homogeneous time-series datasets from real pollination campaigns remain scarce, limiting reproducible modeling, benchmarking, and comparative analysis [7, 8]. To obtain consistent hourly time-series data on colony activity before, during, and after pollination, we monitored nine identical AmoHive smart hives; the hardware architecture and sensor design are described in [9].

This Data Note releases (i) an open seasonal dataset capturing pre-pollination development, a dated pollination service window with a documented intervention event (drone spraying of a bee attractant), and the post-pollination period, and (ii) a fully reproducible data-processing protocol. Together, these resources enable comparative analysis across colonies of readiness for pollination and activity dynamics during pollination and support further research in forecasting, anomaly detection, event-response analysis, and data-quality assessment methods. By enabling evidence-based monitoring of colony readiness and pollination service delivery during short flowering windows, the dataset supports reproducible research and comparative analysis in sustainable agriculture.

## Data description

This dataset comprises time-series of non-invasive IoT measurements collected from nine monitored *Apis mellifera* colonies in Ukraine during the 2024 summer season (01 May–31 Aug 2024; Europe/Kyiv). The record includes a defined sunflower pollination service period (07–23 Jul 2024). An intervention was performed on 10 Jul 2024, when a bee attractant was sprayed by an agricultural drone over the sunflower field hosting hives 027 and 430. Exact apiary coordinates are not disclosed for safety; only region-level information is provided.

Study context and experimental deployment: Throughout the summer season, except during the pollination service period, all nine hives were kept at the home apiary. During sunflower flowering, four colonies (027, 430, 985, 591) were deployed to two sunflower fields ~ 3–4 km apart: treated (027, 430) and untreated (985, 591). The remaining colonies stayed at the home apiary as background controls (286, 883, 224, 448, 841). Prior to deployment, six focal colonies (027, 430, 985, 591, 286, 883) were inspected and balanced for colony strength to improve comparability.

Variables, sensors and raw telemetry: Raw telemetry is provided as nine per-hive CSV exports following the naming convention {hive_id}_01052024_31082024_p.csv. Each file includes a local timestamp (timestamp) and sensor channels for in-hive and ambient temperature (temp1, temp2; °C), in-hive and ambient relative humidity (humid1, humid2; %), hive weight (weight, kg), processor temperature (proctemp, °C), and a stabilized solar voltage (e_uakuv, V). Each AmoHive unit records hive weight and hive microclimate together with near-hive ambient conditions; technical channels support monitoring of device status and power availability.

Cleaned hourly series, provenance labels, and events: In addition to raw exports, the record provides cleaned hourly time series for each hive aligned to a fixed hourly grid (HH:15) in Europe/Kyiv. Raw samples are mapped to the nearest grid tick (± 30 min). The cleaned tables include per-cell provenance columns (*_method) for each numeric channel, distinguishing observed and imputed values. Missing values are imputed using deterministic, rule-based procedures with dedicated handling of sensor artifacts; parameters and transformations are documented in the README, processing protocol, and scripts. Manual beekeeper events are provided as an Excel workbook with one sheet per hive and can be embedded into the cleaned time series.

Validation: Reproducibility is supported by deterministic scripts that convert raw telemetry into cleaned hourly tables with provenance labels. Event timestamps are synchronized with the cleaned series, and a visualization script supports consistency checks.

FAIR compliance: A persistent DOI, data dictionary, standardized fields, access via HTTPS, CSV (UTF-8)/XLSX/MD formats (SI units; ISO 8601 timestamps), CC BY 4.0 for data and MIT for code, checksums, and an event log are provided. Contents are summarized in Table 1.

Reuse potential: The dataset supports (i) pollination-service monitoring studies based on hive weight and microclimate dynamics, (ii) benchmarking time-series forecasting and anomaly detection models, and (iii) comparative evaluation of alignment and imputation strategies using explicit provenance labels, reproducible preprocessing code, and data-quality assessment.

**Table 1:** Overview of data files and datasets

Label	Name of data file/data set	File types (file extension)	Data repository and identifier (DOI or accession number)
Data set 1	1_Pollination_RAW_9_AmoHive_01052024_31082024_p.zip	ZIP (CSV inside)	Zenodo (10.5281/zenodo.17966409) [10]
Data set 2	2_Pollination_Clean_9_AmoHive_01052024_31082024.zip	ZIP (CSV, XLSX inside)	Zenodo (10.5281/zenodo.17966409) [10]
Data file 1	pollination_events_2024.xlsx	XLSX	Zenodo (10.5281/zenodo.17966409) [10]
Data file 2	3_metadata.zip	ZIP (XLSX, PY, MD inside)	Zenodo (10.5281/zenodo.17966409) [10]
Data file 3	README.md	MD	Zenodo (10.5281/zenodo.17966409) [10]
Data file 4	sample_visualization.py	PY	Zenodo (10.5281/zenodo.17966409) [10]
Data file 5	LICENSE_DATA.txt	TXT	Zenodo (10.5281/zenodo.17966409) [10]
Data file 6	LICENSE_CODE.txt	TXT	Zenodo (10.5281/zenodo.17966409) [10]
Data file 7	requirements.txt	TXT	Zenodo (10.5281/zenodo.17966409) [10]
Data file 8	CITATION.cff	CFF	Zenodo (10.5281/zenodo.17966409) [10]
Data file 9	checksums_sha256.txt	TXT	Zenodo (10.5281/zenodo.17966409) [10]
Data file 10	thumb_pol_430_vs_286.jpg	JPG	Zenodo (10.5281/zenodo.17966409) [10]

## Limitations

This dataset is intended primarily for method development and time-series analytics in precision beekeeping rather than for population-level inference or causal evaluation of agronomic outcomes. The record covers a single summer season (May–August 2024) from one smart apiary in Ukraine and includes nine monitored colonies; therefore, generalization across years, regions, and management conditions is limited. Although the dataset contains a defined sunflower flowering window and a documented intervention event, it represents one crop context and one application scheme and does not support broad generalization across multiple crops or farms. The repository does not include standardized, machine-readable agronomic endpoints such as fruit set, seed set, yield, or product quality; consequently, colony telemetry should be interpreted as a proxy for colony activity and colony-level dynamics rather than as a validated indicator of pollination effectiveness. As with many field IoT datasets, telemetry gaps and sensor artifacts may occur due to power availability, connectivity, or environmental factors. While a deterministic cleaning and imputation protocol provides homogeneous hourly series and provenance labels, imputed segments may introduce uncertainty in downstream modeling, particularly for humidity- and weight-derived features.

## Data Availability

The data described in this Data Note are openly available on Zenodo (https://doi.org/10.5281/zenodo.17966409). Details and direct access links are provided in Table 1 and in Reference [10].

## Abbreviations

IoT: Internet of Things

## References

[1] Brewer GJ, Miwa K, Hanford K. Measuring bee effects on seed traits of hybrid sunflower. Plants. 2023;12(14):2662. 10.3390/plants12142662. 37514276 10.3390/plants12142662 PMC10385051

[2] Hauxwell C. Final report: Development of non-invasive methods and systems for the assessment of hive health (PH17001). Hort Innovation; 2024. Available from: https://www.horticulture.com.au/globalassets/laserfiche/assets/project-reports/ph17001/ph17001-final-report.pdf Accessed 16 Jan 2026.

[3] Delaplane KS, Dag A, Danka RG, Freitas BM, Garibaldi LA, Goodwin RM, et al. Standard methods for pollination research with *Apis mellifera*. J Apic Res. 2013;52(4). 10.3896/IBRA.1.52.4.12.

[4] Grant KJ, DeVetter L, Melathopoulos A. Honey bee (*Apis mellifera*) colony strength and its effects on pollination and yield in highbush blueberries (Vaccinium corymbosum). PeerJ. 2021;9:e11634. 10.7717/peerj.11634. 34395063 10.7717/peerj.11634 PMC8323595

[5] Šabić J, Perković T, Šolić P, Šerić L. Buzzing with intelligence: a systematic review of smart beehive technologies. Sens (Basel). 2025;25(17):5359. 10.3390/s25175359. 10.3390/s25175359 PMC12431418 40942788

[6] Kurdin I. Apr. Honey bee diagnostic system in hives. Patent UA133650. 10 2019. Available from: https://patentimages.storage.googleapis.com/84/c4/01/c3c4b02f264ec7/UA133650U.pdf Accessed 16 Jan 2026.

[7] Haddaoui S, Khlifa N, Chikhi S, Varastehpour S, Adjailia F. A comprehensive review of beekeeping datasets for precision apiculture research. In: 2024 10th International Conference on Control, Decision and Information Technologies (CoDIT);, Valletta. Malta. pp. 2043–2048. 10.1109/CoDIT62066.2024.10708308

[8] Kurdin I, Kurdina A. Time-series dataset of non-invasive IoT measurements in *Apis mellifera* colonies: fondant feeding during mild winters and early springs (2018–2020; Ukraine & Canada). Zenodo. 2025. . Accessed 16 Jan 2026. 10.5281/zenodo.18220572

[9] Kurdin I, Kurdina A. Internet of Things smart beehive network: homogeneous data, modeling, and forecasting the honey robbing phenomenon. Inventions. 2025;10:23. 10.3390/inventions10020023.

[10] Kurdin I, Kurdina A. Time-series dataset of non-invasive IoT measurements in *Apis mellifera* colonies: sunflower pollination service during summer season (2024; Ukraine). Zenodo. 2025. . Accessed 16 Jan 2026. 10.5281/zenodo.17966409

